# Immune Activation and Collateral Damage in AIDS Pathogenesis

**DOI:** 10.3389/fimmu.2013.00298

**Published:** 2013-09-26

**Authors:** Frank Miedema, Mette D. Hazenberg, Kiki Tesselaar, Debbie van Baarle, Rob J. de Boer, José A. M. Borghans

**Affiliations:** ^1^Department of Immunology, University Medical Center Utrecht, Utrecht, Netherlands; ^2^Department of Internal Medicine and Hematology, Academic Medical Center, Amsterdam, Netherlands; ^3^Theoretical Biology and Bioinformatics, Utrecht University, Utrecht, Netherlands

**Keywords:** AIDS, pathogenesis, immune activation, TLR, Immunity, therapy

## Abstract

In the past decade, evidence has accumulated that human immunodeficiency virus (HIV)-induced chronic immune activation drives progression to AIDS. Studies among different monkey species have shown that the difference between pathological and non-pathological infection is determined by the response of the immune system to the virus, rather than its cytopathicity. Here we review the current understanding of the various mechanisms driving chronic immune activation in HIV infection, the cell types involved, its effects on HIV-specific immunity, and how persistent inflammation may cause AIDS and the wide spectrum of non-AIDS related pathology. We argue that therapeutic relief of inflammation may be beneficial to delay HIV-disease progression and to reduce non-AIDS related pathological side effects of HIV-induced chronic immune stimulation.

## Chronic Immune Activation is the Primary Driver in HIV Pathogenesis

Upon discovery of the virus that causes AIDS, the name human immunodeficiency virus (HIV) was coined because the virus eventually causes severe immune deficiency. This was based on the clinical symptoms with which end-stage HIV-infected patients presented and on the gradual decline of CD4^+^ T-cell numbers in the blood, which is still considered a hallmark of HIV-disease progression. The finding that HIV is confined to CD4^+^ leukocytes and is cytopathic for CD4^+^ T cells established the hypothesis that HIV causes immune deficiency by directly killing CD4^+^ T cells and impeding CD4^+^ T-cell renewal (1). The molecular mechanisms involved in CD4^+^ T-cell killing by HIV infection have been studied in great detail, leading to novel insights into the down-stream effects of abortive infection and viral integration on cell death (2–4). However, increased apoptosis rates in HIV-infected individuals are not confined to infected CD4^+^ T cells, but are also observed in non-infected CD4^+^ T cells and in cell types that are not even targets for HIV infection, suggesting that the cytopathic effects of HIV are not the full story (5, 6).

Paradoxically, HIV induces strong cellular immune responses, both with respect to magnitude and breadth (7–11), and even in progressive HIV infection, high avidity HIV-specific CD8^+^ T cells are being induced (12). Both CD4^+^ and CD8^+^ T cells are more activated in acute and chronic HIV infection, and hence proliferate rapidly and have a short half life. This explains why both T-cell production and death rates are increased throughout HIV infection (13, 14). At first, the high division rate of CD4^+^ T cells in untreated HIV-infected patients was interpreted to reflect a homeostatic response to the loss of CD4^+^ T cells (15–18). Studies in patients on combination anti-retroviral therapy (cART) pointed out, however, that T-cell proliferation rates drop concomitant with the loss of virus, even when CD4^+^ T-cell numbers are still far below healthy control levels, suggesting that the increased T-cell division rates are caused by the virus itself. It became clear that chronic immune activation is a hallmark of pathogenic HIV infection, exemplified by the increased expression of soluble and cellular immune activation markers, including IFNα, TNFα, and sTNFR and the increased fraction of activated CD8^+^ T cells; markers that have long been used as surrogate markers for HIV-disease progression (19–27). In fact, the level of immune activation is the best predictor of progression to AIDS (28, 29) and death (22, 30–32), independent of HIV viral load. HIV-2 infection is characterized by an overall slower progression rate, lower viral loads, and higher CD4^+^ T-cell numbers than HIV-1 infection (33). Yet, the cytopathicity of HIV-2 for human CD4^+^ lymphoid cells is not lower compared to HIV-1 (34). A striking difference between the two viral subtypes is that the level of immune activation is lower in HIV-2 compared to HIV-1 infection, although expression patterns and prognostic values for immune activation markers were found to be similar when patients with HIV-1 or HIV-2 infection were matched for CD4^+^ T-cell depletion levels (35, 36) These observations were paralleled by insights from simian immunodeficiency virus (SIV) infection in sooty mangabeys (SMs) and African green monkeys (AGMs). SIV infection in these animals is characterized by high viral loads without high levels of immune activation, and does not lead to AIDS, which will be discussed in detail in the Box 1 below (37, 38). Together, these observations have gradually shifted the paradigm from the classical hypothesis that viral cytopathicity is the primary driver of CD4^+^ T-cell depletion and immune deficiency, to the hypothesis that chronic immune activation is the cause of T-cell depletion and immune deficiency (35, 39).

Box 1 **Damage control in non-pathogenic SIV infection.**

In pathogenic SIV infection in rhesus macaques (RMs), high levels of immune activation are associated with progression to AIDS. SIV infection in sooty mangabeys (SMs) and African green monkeys (AGMs), in contrast, do not lead to AIDS despite high viral loads (37, 87–91). Interestingly, SM do not mount stronger cytotoxic T-cell or neutralizing antibody responses to SIV compared to RM, and productively infected CD4^+^ T cells in SIV-infected SM and RM have similar life spans (92–94). Several lines of evidence show that systemically LPS induces features of pathogenic SIV infection (95), that pre-existing microbial translocation and loss of GI integrity in pigtail macaques was associated with faster SIV disease progression (96). In non-pathogenic, like in pathogenic SIV infection, however, a severe depletion of memory T cells in the gut occurs, apparently without causing generalized immune activation in non-pathogenic SIV infection (54, 55).

As the dynamics of virus and virus-infected CD4^+^ T cells in these animal models of SIV infection are comparable, excessive indirect activation-induced killing of T cells in rhesus macaques has been proposed to be the major pathological difference (37, 38, 87, 97–100). Indeed, despite the fact that RM develop strong immune responses upon SIV infection, these responses fail to clear the virus, resulting in persistently high levels of immune activation throughout infection (38, 101).

Compelling evidence has been obtained for a SM-specific polymorphism in TLR signaling, leading to attenuated production of type I IFN by pDCs induced via TLR7/9 activation in SIV-infected SM (102, 103). The gene involved, IRF-7, is a signaling protein downstream of TLR7 and 9. Interestingly, TLR7- and 9-induced production of TNFα appeared to be unaffected in SM, which agrees with the fact that TNFα release is mediated by the NF-κB and not by the IRF-7 pathway. This observation suggests that release of type I IFNs, but not TNFα, may be critical for SIV pathogenesis, which makes IFNα and IRF-7 potential drug targets. Despite the inability of SM to produce high levels of type I IFNs upon TLR7/9 activation by SIV, peak viremia during acute SIV infection in these animals is accompanied by clear signs of an innate and adaptive immune response, including the induction of IFN-stimulated genes (ISGs) (104, 105). Gene expression profiling showed the induction of ISGs, acute inflammatory genes, and genes associated with chemotaxis and neutrophil recruitment, DC activation and maturation, apoptosis, and cytotoxic T-cell responses during the acute phase of both pathogenic and non-pathogenic SIV infection (83, 104–106). In SM and AGM, expression of ISGs returns to normal levels after 30 days of infection. Since this decline in inflammation is paralleled by a gene expression program of immune regulatory genes, including genes that down-regulate T-cell responses [e.g., indolamine 2,3 dioxygenase (IDO), IL-10, LAG3, and PD-L1] and genes that down-regulate IFN responses (e.g., adenosine deaminase), it has been proposed that active downregulation may be involved (83, 104). Further detailed mechanistic studies are required to reveal whether – and if so which – specific down-regulatory pathways are involved. Of note, also host genes implied in intracellular viral restriction are rapidly up-regulated in non-pathogenic infection (83, 104).

If type I IFN is one of the main causes of immune activation in HIV and SIV infection, it remains puzzling how the clear difference in IFNα production by pDC from SIV-infected SM and RM can be reconciled with the apparent similarity of immune responses, and specifically the expression of IFN-inducible genes, observed during acute SIV infection of both species. There is, however, evidence that upregulation of ISGs in acutely SIV-infected SM is induced even though IFNα production by their pDCs is severely diminished (66, 83, 104). Interestingly, Favre and colleagues (66) found upregulation of IFNα, but not IL-12 and IL-6, in acute SIV infection in AGM, although IFNα release was very limited in duration compared to the sustained release of all three cytokines in pathogenic SIV infection. Also the detailed characteristics of immune activation in acutely SIV-infected RM and SM are quite different. Acute SIV infection in SM (and AGM) is not accompanied by increased CD4^+^ T-cell turnover, but strong increases in CD8^+^ T-cell activation, division (Ki67 expression) and apoptosis have been observed (99, 102, 107, 108). Thus, both timing and quality of gene expression of pro-inflammatory cytokines seem to be critically different between pathogenic and non-pathogenic SIV infection (109). Taken together, current data are compatible with the idea that SM and AGM respond to SIV with a limited and transient innate response and with an adaptive response that is mainly restricted to CD8^+^ T cells. In pathogenic SIV infection, an excessive innate response is generated with sustained IFNα and ISG induction which induces proliferation of NK cells and a broad SIV-specific and bystander CD4^+^ and CD8^+^ T-cell response (83, 102, 108). It could be that in SM and AGM, low and transient type I IFN responses during acute SIV infection induce a different gene expression program, allowing for resolution and/or downregulation of the immune response during subsequent chronic SIV infection.

It has been proposed that damage control in SIV-infected SM may in part be due to the preservation of central memory CD4^+^ T cells (T_cm_) which are thought to provide protection against the harmful side effects of bacterial translocation (110). Depletion of memory T cells from the gut and bacterial translocation occur only transiently during acute SIV infection in SMs (54). In contrast to rhesus macaques, SMs are able to avoid epithelial barrier breakdown and thereby limit the undesired side effects of bacterial translocation during chronic SIV infection (111). SM are able to spare T_cm_ from viral infection because of low CCR5 expression (112), while in AGM T_cm_ may be protected against SIV infection by CD4 downregulation (113). In pathogenic SIV and HIV infection, in contrast, T_cm_ are thought to be selectively lost through viral infection (112) However, the observation that the number of activated and naive T cells, and not the number of T_cm_, is predictive for HIV-disease progression does not support the idea that T_cm_ numbers are most critical (114). High levels of immune activation in pathogenic SIV infection may promote SIV infection of T_cm_, resulting in T_cm_ depletion which may contribute to the vicious cycle of loss of immune control. Further investigations are needed to better quantify the contribution of the various mechanisms that cause CD4^+^ T_cm_ activation and death.

In conclusion, non-pathogenic SIV infection of SM and AGM are examples of a pathogen-host symbiosis with an established state of tolerance. This is not immunological tolerance in the strict sense, but a state of tolerance in which the host resists the pathological effects of the virus by avoiding excessive inflammation (115, 116). Further investigation into the various and potentially different mechanisms by which SM and AGM avoid chronic immune activation is warranted and of great importance for our understanding and the treatment of HIV disease.

## Causes of Immune Activation in HIV Infection

It has long been known that innate and adaptive immunity get activated upon acute HIV infection, as extensively described and reviewed elsewhere (35, 39–46). Chronic HIV infection is now known to be characterized by increased expression of pro-inflammatory cytokines, including type I IFNs, IL-6, TGFβ, IL-8, IL-1α, and IL-1β, serum markers of inflammation including sCD14, CRP, cystatin C, D-dimers, and activation of the coagulation system (47). In the last couple of years much attention has focused at the causes of immune activation in HIV infection, with a redirection of research focus from T-cell immunity to innate immunity.

### Breach of gastro-intestinal immunity

In the late 1990s, acute SIV infection in rhesus macaques (RMs) was shown to induce a severe and rapid depletion of memory CD4^+^ T cells from the gut (48). Later, in both humans and monkeys, it was found that this breach of the gut immune system resulted in a significant increase in bacterial components, including lipopolysaccharide (LPS), in the blood (49–51). LPS is a known activator of innate immune cells via Toll-like receptor (TLR) 4, and LPS concentrations in the circulation of HIV-infected individuals correlated strongly with T-cell activation levels (51, 52). It was concluded that translocation of immune stimulatory bacterial products contributes to systemic immune activation, via TLR activation of various leukocyte populations. LPS was used as an indicator for bacterial translocation, but other bacterial products, such as flagellin, peptidoglycan, and bacterial CpG-rich DNA domains that are recognized by TLR2, 5, and 9 respectively, may also contribute to immune activation. It was proposed that the early attack on the memory CD4^+^ T-cell population in the gut may be a critical determinant of disease progression (53). However, also in non-pathogenic SIV infection a severe depletion of memory T cells in the gut occurs, apparently without causing generalized immune activation (54, 55). Moreover, an attenuated variant of pathogenic SIVmac239 was shown to spare mucosal CD4^+^ T cells and yet to cause T-cell activation, CD4^+^ T-cell loss, and progression to AIDS without any signs of microbial translocation (56), showing that immune activation due to gut damage is not required to develop AIDS. On the other hand, in patients on cART, with very low HIV viral load, residual levels of bacterial translocation were positively correlated with immune activation levels suggesting that bacterial translocation may be a dominant driver of immune activation in patients treated with anti-viral drugs (57–63).

The breach of gut integrity in pathogenic SIV and HIV infection has been shown to be associated with depletion of CD4^+^ Th17 cells, a cell type that is normally abundant in the mucosa and is known to be involved in immunity to commensal bacteria (64). It is assumed that the immune system normally keeps a delicate balance between T regulatory (T_reg_) cells and Th17 cells, to protect against pathogens but avoid collateral damage from excessive immune responses (65). The selective loss of Th17 CD4^+^ T cells from the gut – possibly due to selective infection – has therefore been held responsible for the long-term loss of the intestinal integrity and thereby for chronic immune activation in pathogenic HIV infection (64, 66, 67). More recently, depletion of IL-21-producing CD4^+^ T cells has been observed in both the blood and rectal mucosa of SIV-infected RMs (68). Treatment of these animals with IL-21 resulted in the maintenance of intestinal Th17 cells, and a reduction of microbial translocation and systemic inflammation (69). The dynamics of the Th17/T_reg_ balance and the role of Th17 cells and Th17-derived cytokines in HIV infection is currently subject of intensive study.

### Single-stranded RNA, toll-like receptors, and type I IFN production

In 2004 it was reported that TLR7 and 8 recognize RNA from various viruses (70, 71), and it has been demonstrated that single-stranded (ss) HIV RNA directly activates the innate immune system via these TLRs (72, 73). After endosomal binding of ssHIV RNA to TLR7, HIV induces the release of type I interferons by plasmacytoid dendritic cells (pDCs) through the upregulation of TRAIL (72–75). Single stranded HIV RNA has also been shown to activate NK cells in a TLR7 and 8 dependent way, and this process is dependent on cell–cell contact between pDCs and monocytes (76). Finally, pro-inflammatory responses can be induced through intracellular recognition of HIV DNA intermediates. These intermediates can be the result of abortive HIV infection of CD4^+^ T cells, and induce the production of IFN-β and IL-1β (4). In agreement with these *in vitro* observations, gene expression analyses of lymphocytes from HIV-infected persons were shown to have a dominant signature of IFN-stimulated genes (ISGs) (77, 78). Immediately after start of cART – when virus production and viral load rapidly decline – markers of T-cell activation, expression of pro-inflammatory cytokines such as IFNα, IL-6, IL-1-β, and macrophage inflammatory protein-1α, adhesion molecules VCAM-1 and ICAM-1, and the levels of soluble markers for endothelial cell and coagulation activation are all rapidly and strongly reduced, although not to normal levels (15, 18, 73, 79–81). These data suggest that HIV itself, most likely through its ssRNA or DNA intermediates, is an important driver of immune activation in untreated HIV infection.

Type I IFNs provide an important link between chronic innate and adaptive immune activation in HIV infection, because they induce activation and maturation of pDCs, NK cells, T cells, and B cells (82). Gene expression profile data from pathogenic and non-pathogenic SIV-infected primates suggest that persistent release of type I IFNs is a particular feature of pathogenic infection (83). It is well established that pDCs are mass producers of type I IFNs (82). At a certain point, pDCs typically become refractory to restimulation by TLR ligands, thereby avoiding excessive immune activation and collateral damage in the course of viral infection (84, 85). Bhardwaj and colleagues (86) nicely showed that HIV, in contrast to other TLR7 agonists such as influenza virus and herpes simplex virus, induces a partially matured phenotype in pDCs. Because of this phenotype, pDCs are not rendered refractory and continue to produce type I IFNs during ongoing HIV exposure.

Interestingly, and similar to what is observed in SIV-infected SMs (102, 104) and AGMs (83), chronically HIV-infected individuals who do not progress to AIDS despite their high viral loads turned out to have very low levels of proliferating and activated T cells (117) correlating with relatively low levels of ISGs and immune activation gene expression in CD8^+^ T cells (118). A recent study confirmed the central role of IFNα in HIV-1 infection by showing that IFNα is the dominant type I IFN detectable in the plasma of HIV-infected individuals and that its levels correlate with immune activation and depletion of CD4^+^ T cells (119). In addition, it was shown that pDCs derived from women produce more IFNα in response to HIV-1 than pDCs from men, resulting in higher levels of T-cell activation (120, 121). This may at least in part explain the observation that HIV-infected women with a given viral load have a 1.6-fold higher risk to develop AIDS than men, and despite having lower viral loads on average, typically progress faster to AIDS than men (122).

**Figure 1:**
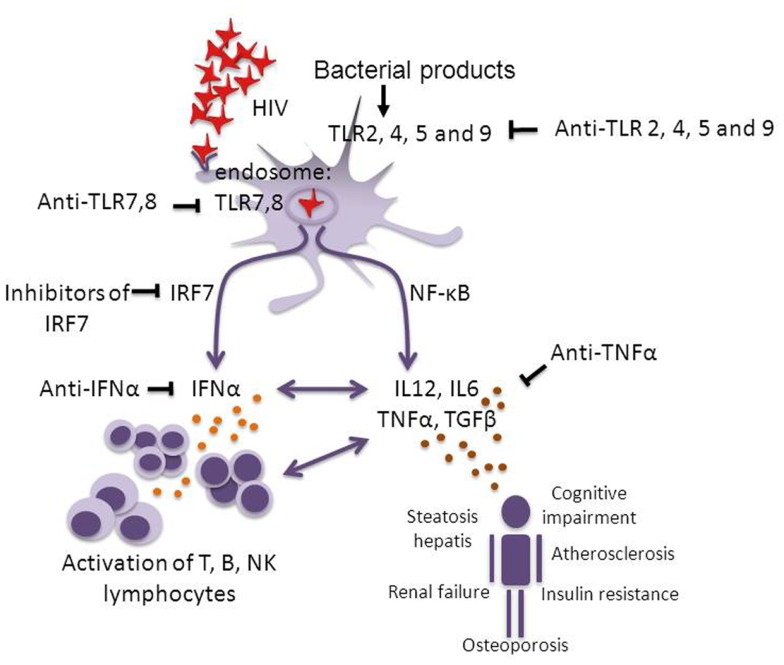
**Pathways of chronic immune activation and its down-stream effects in HIV infection**. HIV infection induces chronic immune activation through activation of the innate and the adaptive immune system, via single-stranded (ss) RNA and possibly through intracellular viral DNA which activate pDCs via endosomal TLR7 and 8. This activation leads to the induction of IFNα via the IRF-7 pathway and the induction of IL-6, IL-12, TNFα, and TGFβ through the NF-κB pathway. Continuous activation of the lymphocyte compartment leads to attrition of the T-cell pool (14, 15) and “immune paralysis” (e.g., impaired CTL responses). Bacterial translocation may be another source of TLR activation via TLR2, 4, 5, and 9 (49–51). Over time also non-AIDS related complications develop. Potential targets for therapeutic interventions with inflammation to diminish pathology are indicated. It has been shown that blocking the effect of TLR7 and 9 significantly reduces HIV-induced immune activation (124). Studies in pathogenic and non-pathogenic SIV infection suggest that blocking IRF-7 or IFNα should be investigated. In rheumatoid arthritis patients who were treated with TNFα inhibiting agents (infliximab, etanercept) it was shown that blocking the effect of TNFα reversed the increased incidence of cardiovascular complications and insulin resistance. In analogy, the potential for a therapy interfering with TNFα in HIV infection should be tested (125–127).

It has been reported that pDCs from SMs have a species-specific inability to produce high levels of type I IFN (102, 103) related to sequence polymorphisms in IRF-7, a signaling protein downstream of TLR7 and 9 (see Box 1). Also in humans, polymorphisms of IRF-7 have been reported that are associated with the level of HIV-induced IFNα production by pDCs *in vitro* and with CD8^+^ T-cell activation *in vivo* (123). These data stress the importance of the IRF-7 pathway in HIV pathogenesis, although there is no definite proof yet that IRF-7 itself is responsible for the induction of different responses in different individuals. Together, these observations suggest that the continuous release of type I IFNs plays a critical role in SIV and HIV pathogenesis. Future studies should point out what the direct and indirect role of IRF-7 polymorphisms is in determining the set point level of chronic immune activation in HIV-infected subjects, and should clarify the potential of IFNα and IRF-7 as drug targets (Figure 1).

## Pathogenic Effects of Immune Activation and Inflammation

The key role of chronic immune activation in HIV and SIV pathogenesis is now commonly accepted, as it is so clearly associated with CD4^+^ T-cell decline and progression to AIDS. The clinical outcome of HIV infection, however, does not only depend on CD4^+^ T-cell loss, but also on non-immunological side effects of chronic immune activation.

### Inflammation drives CD4^+^ T-cell depletion and loss of HIV-specific immunity

A large body of work has suggested that chronic immune activation in HIV infection has deleterious effects on immune function in general, as well as on HIV-specific immunity by inducing persistent activation and maturation of all sorts of innate and adaptive immune cells (82). Through continuous activation and differentiation of T cells, chronic HIV infection gradually depletes the naive CD4^+^ and naive CD8^+^ T-cell pools (31, 35, 43, 128, 129). Intrinsically different responses of the distinct T-cell lineages to activation may determine clonal expansion and contraction (130), and thereby the sensitivity of the different T-cell populations to chronic activation-induced cell loss, although the molecular basis for these differences remains unclear. Thymic and T-cell progenitor dysfunction, most likely caused by aberrantly high levels of pro-inflammatory cytokines expressed during untreated HIV infection, have been reported (43, 131) and the loss of such progenitor cells could aggravate the depleting effects of chronic immune activation on the adaptive immune system. Moreover, continuous inflammation in lymph nodes has been suggested to result in TGFβ-induced collagen deposition, fibrosis, and pathological changes in lymph node architecture, possibly adding to impaired T-cell proliferation and survival (132–134). Continuous activation has recently been shown to induce upregulation of inhibitory receptors such as programed death-1 (PD-1), CTLA-4, and Tim-3, which may interfere with ongoing HIV-specific T-cell responses, and ultimately lead to T-cell anergy and loss of HIV-specific T cells (135–137). Similarly, B-cell dysfunction, which is observed immediately after acute HIV infection (138), is closely related to chronic activation of the B-cell compartment. Increased B-cell turnover and differentiation is associated with the phenotypic and functional B-cell abnormalities characteristic for untreated HIV infection (139–142). A recent study showed the downregulation of the regulatory receptor B- and T-lymphocyte attenuator (BTLA) and the upregulation of PD-1 on B cells in HIV infection (143). Interestingly, a direct down-regulating effect of type I IFN on BTLA expression on CD4^+^ and CD8^+^ T cells has been reported, which may directly contribute to T-cell hyperactivation (144). Recently evidence was reported for a link between PD-1L on follicular Th cells and impairment of B-cells function (145).

Persistent immune activation has also been shown to have deleterious effects on HIV-specific CD4^+^ (7, 146–153) and CD8^+^ T-cell immunity (154–160), amongst others by preventing the establishment of IL-2-producing memory CD4^+^ and CD8^+^ T cells (146, 151–153). HIV-specific cytotoxic T-cell responses are generally considered to play an important role in anti-HIV immunity. Certain HLA alleles clearly correlate with viral load set point and disease progression. In line with this, the major genetic factors related to HIV-1 control coming out of a genome wide association study (GWAS) were shown to affect HLA–viral peptide interaction (161). There is accumulating evidence that Gag-specific CTL responses which preferentially target conserved epitopes have a protective effect (162–171). However, in two large prospective cohort studies, CD4^+^ and CD8^+^ HIV Gag-specific T-cell immunity within the first year after HIV seroconversion were not found to be predictive for disease progression (172, 173). This observation was confirmed in a longitudinal study in an African cohort (174). Also in these studies, immune activation turned out to be the strongest risk factor for disease progression, stronger than, and independent of, viral load (172, 173). It is important to consider the possibility that the typical association between strong CTL responses and a lack of HIV-disease progression that is observed in cross-sectional studies, may merely reflect the preservation of CTL responses in the absence of chronic immune activation rather than a protective effect of CTL themselves (175).

### HIV-induced inflammation and HIV-associated non-AIDS disease

Increasing insight in the source and the role of inflammation in HIV pathogenesis has been paralleled by recent progress in our understanding of the role of inflammation in a much wider spectrum of clinical conditions than infectious diseases. After the introduction of anti-retroviral therapy for HIV infection, several case studies suggested that patients treated with cART had an increased risk to develop sub-clinical atherosclerosis and acute myocardial infarction (176–179). Initial studies reported that the increased risk of cardiovascular disease was associated with specific classes of anti-viral drugs (180). Later studies revealed that cardiovascular risk was in fact larger in untreated compared to treated HIV infection (181, 182), but also in patients on cART, the risk for cardiovascular disease is higher than expected based on traditional cardiovascular risk factors alone. In addition to cardiovascular disease, HIV infection poses patients at increased risk to develop a number of other non-AIDS related complications, such as non-alcoholic steatohepatitis, renal dysfunction, osteoporosis, insulin resistance, metabolic syndrome, and cognitive impairment (47). It has been shown that soluble mediators released by activated immune cells, such as IL-6, IL-1, and TNFα, also act on non-immune tissue cells with various tissue-dependent pathological effects. In a broad variety of clinical conditions, including obesity, atherosclerosis, neurodegenerative disease, and autoimmune diseases, chronic inflammatory processes are now recognized to play a major role (183), and it has been postulated that most non-AIDS defining complications of HIV infection are related to the chronic inflammatory state induced by HIV (Figure 1) (184, 185). This hypothesis is strengthened by recent observations in patients with rheumatoid arthritis (RA). Both HIV infection and RA are characterized by a chronic inflammatory state and increased levels of pro-inflammatory cytokines like TNFα, IL-1β, and IL-6, and also in RA patients the incidence of non-primary disease related complications such as cardiovascular disease, osteoporosis, non-alcoholic fatty liver disease (NAFLD), and cognitive impairment are more prevalent than among the general population (125–127). Thus, clinical symptoms that initially seemed unrelated are now being recognized as part of the total complex of HIV-associated disease and appear to have a common underlying pathogenesis of chronic inflammation and excessive immune activation (186, 187). Preliminary data suggest a central role for TNFα in HIV-associated non-AIDS disease but it remains to be determined to what extent other pro-inflammatory cytokines, perhaps acting via TNFα, are involved.

## HIV in Comparison to Other Persistent Viral Infections

These novel insights into HIV pathogenesis prompt the question as to how HIV differs from most other viruses. We believe that HIV pathogenesis is caused by a combination of specific characteristics. Most importantly HIV infects CD4^+^ T helper cells. In addition a variety of cells that express CD4 and one of the HIV coreceptors can be infected albeit at very low levels. Thereby, the virus is not confined to a single organ and may induce a variety of systemic immune responses. HIV induces much higher levels of cytokines during acute infection compared to hepatitis B or hepatitis C (41). HIV is virtually insensitive to control by neutralizing antibodies and cellular immunity because of various mechanisms, including the glycan shield surrounding the HIV virion (188) and the high mutation rate of the virus, which allows for rapid immune escape. After acute HIV infection, virus- and host-specific set points are established that determine the subsequent clinical course based on the level, and probably the type, of immune activation that is induced. Like other viruses, HIV induces type I IFN release by pDCs. The fact that HIV is targeted to pDCs by virtue of their expression of CD4, and the recent finding that HIV does not induce full maturation of pDC, which prevents these cells to become refractory to restimulation, as outlined above (86), may turn out to be critical factors driving persistent IFN release and thereby chronic activation of the innate and the adaptive immune system in HIV patients, resulting in exhaustion of immunity and broad spectrum end-organ immune pathology. Even though immune responses in acute hepatitis B and hepatitis C virus infection may differ from those in acute HIV infection, in individuals who do not clear hepatitis viral infection and who convert to chronic hepatitis, persistently increased immune activation levels have been reported. In analogy to what is observed in HIV patients, also non-hepatitis related conditions, such as metabolic syndrome and cardiovascular disease, occur more frequently in chronic hepatitis patients than in the general population, even when corrected for traditional risk factors for, e.g., cardiovascular disease (189). Strikingly, peripheral blood naive T-cell numbers in chronic hepatitis C virus-infected patients were found to be significantly lower than in healthy individuals, and associated with increased levels of inflammation (190). Thus, while immune responses during acute infection may differ between HIV, hepatitis B, and hepatitis C virus infection, leading to clearance of the virus in the majority of hepatitis B infected patients and a subset of hepatitis C infected individuals, once chronic inflammation has been established, its effects tend to be similar for the three patient groups. Other viral infections, like Epstein–Barr virus (EBV) and cytomegalovirus (CMV) are incomparable to HIV or chronic hepatitis infection, because after an acute phase these infections convert into a truly latent stage, during which no virus is detectable in the peripheral blood of immunocompetent individuals.

## The Immune Activation Hypothesis Reduced to Practice

### Boosting immunity

Great effort has been put over the years into approaches to therapeutically strengthen anti-viral immune responses. Thus far, however, there is little proof for beneficial effects, and in fact the possibility of induction of adverse effects is an important concern. Therapeutic vaccination, with DNA and live viral vector based vaccines and combinations thereof, has had only transient and small effects on viral load (191, 192). In one trial in which therapeutic vaccination was followed by interruption of cART, viral rebound was larger and time to restart therapy shorter, than in the non-vaccinated group (193). With respect to prophylactic vaccines, CTL-based vaccines may have some potential if they manage to consistently lower the viral set point. However, upon infection such vaccines will at best reduce and not completely prevent chronic immune activation driven pathogenesis and should therefore not be considered as curative. In fact, the strongest protective effect is to be expected from HIV vaccines that stimulate HLA-B57, B58, or B27 restricted T-cell responses, as they are associated with significantly lower viral loads. Such vaccines would however only help the carriers of protective HLA molecules, most of which already experience much slower disease progression upon HIV infection. In order to develop CTL vaccines that are applicable to a wider patient population it is of vital importance to gain better insight into the mechanisms responsible for the relative protection conferred by these protective HLA molecules.

For boosting of immunity and enhancement of CD4^+^ T-cell production, IL-2 has been administered in large scale multi-center international trials in patients with and without cART with substantial increases in CD4^+^ T-cell counts but no beneficial clinical effects (194). Administration of IL-7 (195, 196) or human growth hormone (197) has been tried out in small cohorts, with successful effects on naive and central memory T-cell numbers, but again without significant clinical effects. As these biological compounds are known to have strong activating effects on the peripheral T-cell compartment (198) their administration is not without risk, and one should be aware of possible adverse effects in the long run. Immune stimulating therapy should in any case be restricted to patients on cART, although even on cART (residual) immune activation is correlated with poor immune reconstitution (199). To enhance anti-HIV responses, blockade of inhibitory ligand-receptor interactions, such as PD-1, CTLA-4, and Tim-3, has been proposed (200). Some positive results have been obtained with PD-1 blockade in SIV-infected macaques, which has been shown to lead to improved virus-specific CD8^+^ T-cell responses, reduction in plasma viral load and prolonged survival (201), and to reduced hyperactivation and bacterial translocation (202). However, experiments with CTLA-4 blockade have demonstrated that the effects of inhibitory receptor blockade may even be deleterious, leading to increased T-cell activation and viral replication (203). Great care therefore needs to be taken with approaches that may increase the level of CD4^+^ T-cell proliferation, and in our opinion should never be applied without cART.

Taken together, therapeutic interventions aiming at enhancing anti-HIV T-cell immunity may not have the desired beneficial effect in the majority of people, and may even have adverse long-term effects through the immune stimulation they induce. It has been argued that since our understanding of virus-specific cellular immunity – and in particular its repertoire, its functional and kinetic requirements, and its regulation and tissue distribution – are still far from complete, the real correlate of immune protection against AIDS is still to be discovered (204). Indeed, not all immune activation needs to be equally pathogenic, and we cannot exclude the possibility that induction of HIV-specific T-cell responses *without* excessive and chronic release of type I IFNs and other cytokines might be favorable to the host for control of HIV. However, as pDC activation is believed to be required for the induction of an adequate adaptive T-cell response, induction of strong HIV-specific immune responses without chronic release of type I IFNs may be an impossible combination; in fact, pDC activation may collaterally cause the very same pathology that the adaptive immune response should prevent. Irrespective of the hypothesis of what is causing AIDS pathogenesis, of all vaccination strategies, prophylactic vaccines that are able to induce a strong broadly neutralizing antibody response at this time seem to be most promising to induce protective immunity to HIV infection in a large number of individuals (205).

### Therapeutic damage control

Another correlate of the immune activation hypothesis is that immune suppressive therapy might have beneficial clinical effects because it reduces the deleterious effects of immune activation. Immune suppressive drugs like cyclosporin (206, 207) and mycophenolic acid (208), that are used to prevent T-cell activation in organ transplant rejection, have been experimentally tried in HIV infection. In combination with cART, variable effects on T-cell turnover, activation, and CD4^+^ T-cell numbers were shown (206–208).

Given the recent insight that not activation of CD4^+^ and CD8^+^ T cells via TCR, but instead TLR activation, release of type I IFNs and expression of IFNα/β inducible genes may contribute more to systemic immune activation in HIV infection, the latter proteins and genes may be more relevant targets for therapeutic interventions (Figure 1). TLR antagonists and inhibitors are currently an area of intense investigation and it is to be expected that many will become available for phase I/II or experimental proof of concept clinical trials in the very near future (209, 210). Indeed, in a preliminary study in which chloroquine, an inhibitor of endosomal TLR3, 7, 8, and 9 was administered to HAART-naive HIV-infected patients, significantly lower immune activation levels were observed, as reflected by decreased levels of T-cell division and expression of activation markers (124). Although these findings need to be reconfirmed and more clinical studies are needed, this study suggests that interference with HIV-induced TLR7/9 activation is feasible. Because of the clear association between immune activation and clinical outcome such interventions may be promising. Also IRF-7, which selectively induces IFNα but not TNFα or IL-12 production, is a potential drug target and treatment with IFNα neutralizing antibodies or blocking TNFα or the TNFα-R are feasible options to be explored in order to decrease inflammation and tissue-related pathology. Indeed, targeting TNFα in pathogenic SIV infection in RMs by administration of adalimumab (Humira) has been shown to reduce systemic inflammation and many of its down-stream effects (211).

Humanized anti-IFNα monoclonal antibodies have been developed and have been tested in phase I trials in patients suffering from systemic lupus erythematosus (SLE) and psoriasis, autoimmune diseases in which IFNα is believed to play a critical role. In SLE but not psoriasis one dose of anti-IFNα monoclonal antibody resulted in downregulation of IFN-inducible gene expression with beneficial clinical effects (212, 213). No evidence for adverse effects, such as an increase in viral infections or viral reactivation was observed which opens up the possibility to consider application of anti-IFNα treatment to HIV-infected patients to neutralize over-expression of IFNα. Induction of anti-IFNα antibodies by immunization with inactivated IFNα to inhibit progression to AIDS has been investigated in a large multicentre study, and beneficial effects on CD4^+^ T-cell decline and markers of clinical progression were reported in patients that developed anti-IFNα antibodies (214). Although these studies have never been repeated, the recently obtained insights into the role of IFNα in HIV-disease progression warrant future research in this direction.

Paradoxically, IFNα administration has been investigated in the pre-cART era as a treatment option for HIV infection with or without Kaposi sarcoma (215, 216). Although IFNα treatment showed the expected anti-viral effect, leading to lower viral loads, this type of treatment became of less interest when cART became available. In addition, IFNα treatment induced flu-like syndrome, immune activation, and T-cell depletion when given to HIV patients co-infected with HCV (217–219).

In RA patients who were treated with TNFα inhibiting agents (such as infliximab or etanercept) it was shown that blocking the effect of TNFα reversed the increased incidence of cardiovascular complications and insulin resistance (125–127). Anecdotal reports have shown the safety of anti-TNFα treatment in RA patients who were also HIV infected and on HAART (220). A non-specific intervention aimed at lowering immune activation and its side effects, such as cardiovascular disease, might be the addition of statins to standard anti-retroviral regimens, as has been suggested for treatment of RA (221).

In addition to potentially improving HIV-treatment options, the interventions suggested above will provide us with a wealth of data allowing dissection of the relative contribution of different cytokines such as IFNα and TNFα to immune activation and end-organ immune pathology in HIV infection. It should be noted however that, given the complex interrelationship between potentially protective immune responses and the damage induced by chronic immune activation, any of these interventions could in principle also aggravate HIV-induced pathology. Therefore, a combination with HAART seems at this time the best approach.

## Conclusion

We review compelling evidence for CD4^+^ T-cell loss in HIV infection caused by various down-stream effects of persistent and strong innate immune activation. Immune activation is induced by HIV ssRNA and possibly its DNA intermediates and to some extent by translocation of bacterial products from the gut. This CD4^+^ T-cell death is occurring in addition to CD4^+^ T-cell loss due to direct HIV-induced cell killing. We conclude that immune activation is most likely the main cause of CD4^+^ T-cell depletion, loss of HIV-specific immunity and HIV-associated non-AIDS disease, also in patients on cART. Although much knowledge is still lacking, we are beginning to understand which receptors and active molecules are most likely dominant in the cellular and molecular pathways involved in HIV pathology. This new perspective has major implications for HIV vaccinology, but also opens up novel therapeutic options that may be explored in the near future.

Search strategy and selection criteria: references for this article were identified through searches of PubMed for articles published from 1985, by use of the terms HIV, SIV, AIDS, immune activation, immunity, pathogenesis. Articles resulting from these searches and relevant references cited in those articles were reviewed. Articles published in English were included.

## Conflict of Interest Statement

The authors declare that the research was conducted in the absence of any commercial or financial relationships that could be construed as a potential conflict of interest.
